# Evaluation of the Genotoxic Effects of Grape Seed Extract and Marine Collagen Peptide on the Fibroblast Cell Line: An In Vitro Study

**DOI:** 10.7759/cureus.61605

**Published:** 2024-06-03

**Authors:** Neshkumar KLS, Kavitha Ramar

**Affiliations:** 1 Pediatric and Preventive Dentistry, SRM Kattankulathur Dental College, Chennai, IND; 2 Pedodontics and Preventive Dentistry, SRM Kattankulathur Dental College, Chennai, IND

**Keywords:** dapi staining, fibroblast cell line, genotoxicity, peptides, grape seed extract

## Abstract

Introduction

Collagen plays a vital role in maintaining the structural integrity of dentin, and its modification with bioactive compounds can enhance its mechanical properties and bonding capabilities.

Aim

This study aimed to evaluate the genotoxic effects of grape seed extract (GSE) and marine collagen peptide (MCP) on dental pulp-derived primary cells.

Methodology

Human dental pulp stem cells were isolated, cultivated, and then treated with GSE and marine collagen peptides. DNA fragmentation was assessed using DAPI (4′,6-diamidino-2-phenylindole) staining. Statistical analysis was performed using SPSS version 20 (IBM Corp., Armonk, NY, USA).

Results

The results showed that GSE exhibited a minimum level of cell death compared to marine collagen peptides. The viable cell count increased steadily over three days in all groups, with the control group showing the highest number of viable cells. The differences in viable cell count among the groups were statistically significant.

Conclusion

This study suggests that GSE and marine collagen peptides are highly biocompatible with dental pulp cells and could be considered for further clinical studies.

## Introduction

Collagen is essential for the maintenance of a cytoskeleton and is a major component of extracellular matrix proteins in multicellular organisms such as humans [1]. An effective method for enhancing dentin's mechanical, anti-enzymatic, and bonding capabilities is collagen bio-modification mediated by bioactive compounds.

Collagen molecules get their tensile strength from intracellular cross-links. The collagen fibers that are vulnerable to enzyme destruction are exposed during dentin demineralization. Colonizing cariogenic bacteria use the demineralized dentin collagen as a framework. Proteolytic enzymes degrade the exposed collagen and the structural integrity of collagen fibers during later stages of breakdown. According to Tjäderhane et al. (2015), host-derived collagenolytic enzymes can destroy an exposed telopeptide region of the collagen molecule during an early stage of demineralization, causing collagen to lose its cross-banding [2]. Extra inter- and intramolecular cross-links can be induced by using collagen cross-linking chemicals that are not naturally present in the material. The use of selective cross-linking agents has been shown to enhance demineralized dentin's ultimate tensile strength and elastic modulus. Fruits, vegetables, nuts, seeds, and flowers are abundant sources of proanthocyanidin (PA), a powerful cross-linking agent with antioxidant and biological effects [3]. Recent research has demonstrated that demineralized dentin can have its mechanical qualities enhanced by utilizing a grape seed extract, which is primarily constituted of PA. Conventional wisdom holds that a "hybrid layer" formed when adhesive monomers fully enter and envelop exposed collagen fibrils yields the best results when bonding to dentin [4]. Hydrolysis and enzymatic degradation of exposed collagen and an adhesive resin are two of the physical and chemical mechanisms that contribute to the hybrid layer's deterioration [5-7]. Some have hypothesized that water-filled canals grow within the hybrid layer, as its components start to degrade. The possibility of further deterioration is increased since these canals permit oral and dentinal fluids to reach the hybrid layer [8]. Recent research indicates that collagen peptides produced from the host play a crucial role in the breakdown of the hybrid layer while bacterial enzymes may also be involved. The creation of a hybrid layer involving the interlocking of micromechanical elements between demineralized collagen fibrils and infiltrated methacrylate adhesive resin is essential for resin-dentin bonding. One of the most crucial aspects of dental restoration success is the bio-stability of the underlying mineralized collagen. Grape seed extract and marine collage peptide are naturally available products, the main aim of this study is to evaluate the genotoxic effects of grape seed extract (GSE) and marine collagen peptide (MCP) on dental pulpal stem cells. The main objective of the study is to evaluate the invitro genotoxicity effect of natural materials by seeing the extent of DNA damage. The null hypothesis states that there will not be any cytotoxic and genotoxic changes in both materials.

## Materials and methods

Human dental pulp stem cell (DPSC) isolation and cultivation

The extracted teeth were transported to the laboratory in Dulbecco’s Modification of Eagle’s Medium (DMEM) supplemented with 10% fetal calf serum (FCS), 200 units/mL penicillin, 200 pm/mL streptomycin, 2 mmoll/L glutamine, non-essential amino acids, and 20 pg/mL fungizone (explant medium). Once in the laboratory, each tooth was wrapped in a rubber dam and placed in a vice. The vice was tightened until the tooth cracked open. The pulp could then be removed easily. The extirpated pulp was dissected into thirds and only the middle third was kept and used for subsequent cell isolation. This portion of the pulp was finely minced and placed into 25 cm^2^ tissue culture flasks previously treated with an explant medium to facilitate the binding of the tissue portions. The explants were then incubated at 37°C in a humidified atmosphere of 95% air and 5% carbon dioxide (CO_2_). The explant medium was gradually changed to a standard culture medium containing 10% FCS, 100 units ml penicillin, 100 pg/mL streptomycin, 2 mmol/L glutamine, and non-essential amino acids. The explants were examined daily and notes were made concerning the outgrowth of cells and the time taken to reach a radius of 5 mm. Fibroblast cell lines were established by subsequent trypsinization and culturing through several passages. Only those cells between the fourth and ninth transfers in culture were used for subsequent analyses.

Grouping

The obtained cultured dental pulpal cells were seeded into 6 well plates (90000) cells/well in its media 2 ml/well and divided into 3 groups. Three wells must be prepared as technical replicates for each group and they will incubated for 24 hours.

Group 1 cells were the control group where no treatment was done with any materials. Group 2 (6 wells/triplicate) was treated with 10 microgram ml of GSE extract and group 3 was treated with 10 microgram/ml of peptides extract. The experiments’ biological triplicates were carried out by repeating the same protocol using the three sets at three separate times to prove reproducibility.

Evaluation of DNA fragmentation by DAPI (4',6-diamidino-2-phenylindole) staining

The biocompatibility and DNA integrity of dental pulp-derived primary cells of control and experimental groups were determined by DAPI staining. At a density of 5x10^4^ cells/well, cells were seeded into 24-well plates and subjected to treatment with either the control or experimental groups for 24, 48, or 72 hours. After 30 minutes of fixation in 4% paraformaldehyde in phosphate-buffered saline (PBS) at room temperature, the cells were permeabilized with 0.25% Triton X-100 (Sigma-Aldrich, St. Louis, Missouri, USA) in PBS for 30 minutes. Finally, they were stained with DAPI for 4 minutes in the dark. The unstained and stained cells were observed under phase contrast inverted fluorescence microscopy (Invitrogen, Evos, Fisher Scientific, Hampton, New Hampshire, USA).

Statistical analysis

Data regarding the number of viable cells in live/dead assays and DAPI staining in the control, GSE, and peptide groups on days 1, 2, and 3 were entered into Microsoft Excel (Microsoft Corporation, Redmond, WA, USA)and analyzed using IBM SPSS Statistics for Windows, Version 20 (IBM Corp., Armonk, NY, USA). Data were investigated for normality using the Shapiro-Wilk test and assessed for normal distribution of both variables. Continuous variables were presented as mean ± standard deviation (SD) and 95% confidence interval. Two-way ANOVA was used to analyze the difference in the mean number of viable cells in DAPI staining in three groups over three days followed by post hoc analysis using adjusted Bonferroni multiple pairwise comparisons. The level of significance was determined at p≤0.05.

## Results

DAPI staining

In this study, DAPI staining was utilized as a method to assess genotoxicity, particularly focusing on cells with varying proportions of AT-rich DNA. DAPI (4',6-diamidino-2-phenylindole) is known to preferentially bind to regions of DNA that have a high adenine-thymine (AT) content. This staining method allows for the visualization and quantification of DNA damage or alterations in DNA structure, which can be indicative of genotoxic effects.

The experimental procedure involved treating cells with either grape seed extract or marine collagen peptide for a duration of three days. Following treatment, the cells were subjected to DAPI staining. The addition of a phosphate solution served to raise the pH, creating a buffer system conducive to DAPI staining. Under these conditions, DAPI acts as an effective and specific DNA stain, allowing for the visualization of DNA within the cells.

Subsequent examination of the stained cells under a fluorescent microscope revealed notable differences between the two experimental groups. Specifically, cells treated with grape seed extract exhibited a minimal level of cell death compared to those treated with marine collagen peptide. This observation suggests that grape seed extract may have a protective effect against genotoxicity, as evidenced by the lower incidence of cell death observed in these cells.

The differential response of the cells to the two treatments highlights the potential differences in their genotoxic effects. While grape seed extract appears to confer some level of protection against DNA damage or cell death, the marine collagen peptide treatment may be associated with a higher degree of genotoxicity. Further analysis and characterization of these effects could provide valuable insights into the mechanisms underlying the observed differences and the potential implications for health and safety considerations.

**Figure 1:**
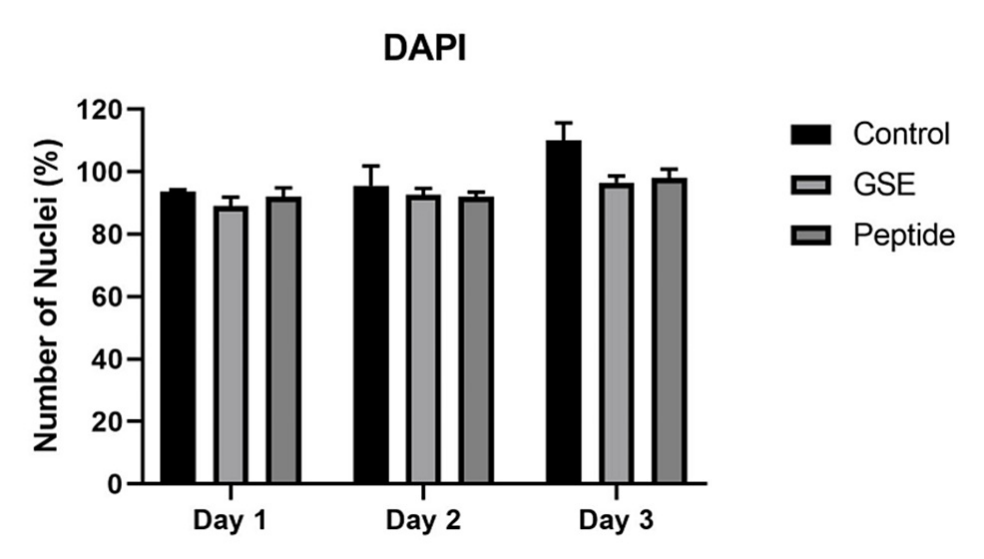
Represents the number of nuclei (percentage) treated with GSE and peptide GSE: grape seed extract

**Figure 2:**
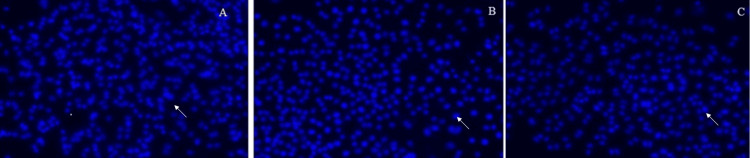
Day 1: 2A represents the control group; 2B represents the cells treated with grape seed extract; 2C represents the cells treated with marine collagen peptide The arrows indicate the pulpal cells after the DAPI (4′,6-diamidino-2-phenylindole) staining method for the extent of DNA damage of three groups on Day 1.

**Figure 3:**
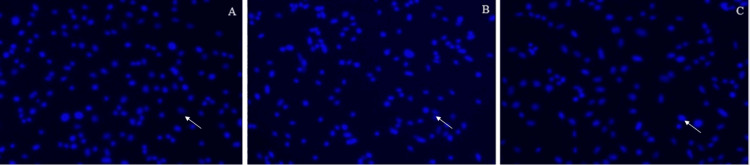
Day 2: 3A represents the control group; 3B represents the cells treated with grape seed extract; 3C represents the cells treated with marine collagen peptide The arrow indicates the pulpal cells after the DAPI (4′,6-diamidino-2-phenylindole) staining method for the extent of DNA damage of three groups on Day 2.

**Figure 4:**
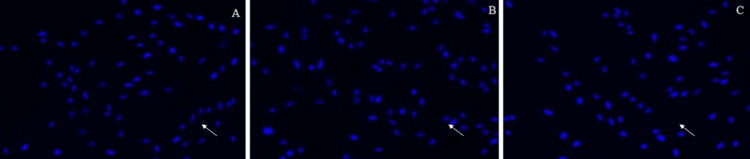
Day 3: 4A represents the control group; 4B represents the cells treated with grape seed extract; 4C represents the cells treated with marine collagen peptide The arrow indicates the pulpal cells after the DAPI (4′,6-diamidino-2-phenylindole) staining method for the extent of DNA damage of three groups on Day 3.

The number of nuclei (percentage) after being treated with GSE and peptide is represented in Figure 1. The extent of DNA damage is represented in Figures 2-4.

The biocompatibility and DNA integrity of dental pulp-derived primary cells by the control and experimental groups were determined by DAPI staining. DAPI staining shows that the mean viable cell count in the control group was 94.00 (1.00) on day 1, 95.66 (4.50) on day 2, and 109.33 (4.16) on day 3. In the GSE group, the mean number of viable cells on day 1 was 88.66 (2.08), on day 2 was 92.33 (1.52), and on day 3 was 92.66 (1.52). The peptide group showed the mean viable cell count on day 1, day 2, and day 3 was 93.00 (2.64), 92.66 (1.52), and 98.33 (2.08), respectively.

The viable cell count was amplified slowly and steadily over three days in every group. The control group showed the highest number of viable cells in total and over three days. The least number of viable cells was observed in the GSE group and with the peptide group on day 1. GSE on day 2 and day 3 showed a similar number of viable cells to peptide. The differences between the mean number of viable cells among each group (control, GSE, and peptide: F value = 17.614, p = 0.000); days effect (1, 2, and 3: F value = 34.408, p-value = 0.000) and staining effect among groups for the complete duration (F value = 4.130, p = 0.015) was found to be statistically significant. The adjusted Bonferroni test for multiple pairwise comparisons for groups shows that the mean difference between the control and GSE (MD = 7.111, p = 0.000) and control and peptide (MD = 5.000, p = 0.002) is statistically significant (Table 1). This suggests that the GSE and peptide groups have a comparable effect on the count of viable cells with DAPI staining.

**Table 1:** DAPI staining (count of viable cells): adjusted Bonferroni test for multiple pairwise comparisons for groups *Mean difference is significant at the .05 level Two-way ANOVA was used to analyze the difference in the mean number of viable cells. The level of significance was determined at p≤0.05. DAPI: 4′,6-diamidino-2-phenylindole; ANOVA: analysis of variance

DAPI staining (count of viable cells): adjusted Bonferroni test for multiple pairwise comparisons for groups						
(I) Groups	(J) Groups	Mean Difference (I-J)	Std. Error	p-value		
					Control	GSE	7.111^*^	1.231	0.000		
Peptide	5.000^*^	1.231	0.002			
GSE	Control	-7.111^*^	1.231	0.000		
	Peptide	-2.111	1.231	0.310		
Peptide	Control	-5.000^*^	1.231	0.002		
	GSE	2.111	1.231	0.310		

## Discussion

Various naturally occurring fruits, food supplements, and plant extracts have lately demonstrated their ability to improve general health, and it has been proposed that antibacterial chemicals derived from these plants may be an alternative to routinely used synthetic drugs [9]. The GSE is one such natural substance [10,11]. By co-polymerizing with adhesive monomers, the PAs created in this study may represent a novel class of polymerizable collagen crosslinkers with the ability to crosslink dentin collagen to strengthen it while also building a strong bond with adhesive resin [12]. PAs made from grape seed extract contain 70-80% oligomers of flavonoids. Although the chemical makeup of their flavonoid building blocks may be complex, catechins, epicatechins, and their gallate derivatives predominate [13].

When it comes to dental health, a wide variety of biomaterials are already used in clinical applications. When determining if biomaterials are compatible with living organisms, it is essential to measure their cytotoxicity [14]. While it's true that natural products often don't have any noticeable local side effects, it's also common knowledge that they might have negative impacts on overall health. Because they are more stable and require less attention when cultured, fibroblast cell lines are a popular alternative to primary cells [15].

It is ideal for therapeutic materials to have a desired effect while simultaneously minimizing or eliminating cytotoxic effects and preserving maximum tissue viability.

To establish initial biocompatibility, this study compared the two materials with periodontal ligament fibroblasts by measuring cell adhesion as measured by the creation of focal contacts. Since immunocytochemistry is a specialized test for detecting Integrin β1 and Vinculin, it was used to analyze this parameter. As an adhesion molecule, vinculin is surface-localized on cell membranes and, among other things, acts as an "anchor" to start and sustain fibroblast attachment to the surface. Integrin β1 is a transmembrane molecule that complements cell-surface adhesion by acting as a "binder" of extracellular matrix components like fibronectin, collagen, and vitronectin. The results showed that the GSE-treated group had the lowest Vinculin expression, according to the photographs. Cell death was less in both groups, proving that it had less cytotoxicity. Escobar Garcia et al. (2016) found that cytotoxicity and cellular adhesion of mineral trioxide aggregate (MTA) and Biodentine (BD) on periodontal ligament fibroblasts (PDL) and concluded that neither material was cytotoxic during the time evaluated. The DNA dye shows a fluorescence boost of approximately 20 times when bound to AT sections of double-stranded DNA while grape seed extract and marine peptides both increase and induce cell death through simultaneous chromatin condensation and DNA breakage. Genomic staining, fluorescence microscopy, and flow cytometry all make use of this dye, which is activated by the violet (405 nm) laser line [16]. The peptide group showed much more DNA fragmentation than the GSE group in this investigation. The number of nuclei present in both groups remains the same after the procedure is done in triplets. Similarly, an Enterococcus faecalis (E. faecalis) biofilm model was used by Maria Dede et al. to assess the efficacy of endodontic disinfection protocols using DAPI staining and analyze bacterial viability; it found that the irrigants exhibit different cytotoxic levels by the combination of 3% NaOCl with 20% EDTA. Every biomaterial needs to undergo in vitro biocompatibility tests, which are crucial instruments in the field of dentistry [17].

Materials derived from natural sources may also have less of the unpolymerized components that could be harmful. A possible explanation for the low levels of cytotoxicity and genotoxicity found in the present study could be acceptable.

Limitations

Genotoxicity studies are often conducted in vitro using cell culture models, which may not fully recapitulate the complex microenvironment and physiological conditions found in vivo. Many genotoxic agents require metabolic activation by cytochrome P450 enzymes or other metabolic pathways to exert their genotoxic effects. In vitro cell culture systems may lack the necessary metabolic capacity, leading to underestimation of genotoxicity. Including metabolic activation systems or conducting studies in metabolically competent cell lines or animal models can help address this limitation.

## Conclusions

Natural agents adjunct to other fluoridated and non-fluoridated synthetic remineralizing materials and promising non-invasive therapy for carious lesions in primary teeth. GSE combined with peptide and colophony can be incorporated into various other products like toothpaste, gel, and varnish and can be applicable in clinical conditions in place of or as an adjuvant to the topical application of fluoride. The GSE and peptide-treated human dental pulp cells were highly biocompatible, which was assessed by DAPI staining to observe the DNA integrity with untreated control cells. Overall, the in vitro evaluation suggested that the GSE and peptide can be considered to move for clinical study after confirming its biocompatibility in the in-vivo model.
